# Rapid generation of an RBL cellular model to study proteins that cause allergenic reactions in vitro

**DOI:** 10.1007/s12026-024-09461-0

**Published:** 2024-02-09

**Authors:** Israel Hernández-Aguilar, Juan Carlos Vizuet-de-Rueda, Miguel Ángel Galván-Morales, Josaphat Miguel Montero-Vargas, Luis M. Teran

**Affiliations:** 1 Ciencias Básicas en Biología, Instituto Tecnológico del Valle de Oaxaca, Ex-Hda. de Nazareno, Santa Cruz Xoxocotlán, 10587 Oaxaca, México; 2 Depto. de Inmunogenética y Alergia, Instituto Nacional de Enfermedades Respiratorias Ismael Cosío Villegas, Calz. de Tlalpan 4502, Tlapan, 14080 Ciudad de Mexico, México

**Keywords:** RBL, Aeroallergens, Diagnostic, Allergens

## Abstract

Allergic diseases affect nearly 30% of people worldwide. There is a wide range of allergen sources, such as animal dander, food, venom, dust mites, and pollen. The skin prick test is the predominant technique used to identify allergenic sensitivity in vivo; the main problem is that it can be imprecise as many of the allergen extracts are made of mixtures of allergic and nonallergic components, making it difficult to identify the disease-eliciting allergen. An alternative to solve this problem is employing cellular models in vitro that may allow allergen identification, allergy diagnosis, and testing of novel potential compounds that can be used in immunotherapeutics. For example, rat basophilic leukemia (RBL) cells are a well-suited model for studying allergies. Unfortunately, cells generated from RBL cells are not commercially available. Therefore, we developed an RBL model with a degranulation gene reporter capable of recognizing human IgE involved in allergenic sensitivity using commercial plasmids. Employing this model, we successfully evaluated the capacity of union between IgE from allergic patients to allergenic proteins from Oleaceae tree pollen. This RBL cell model can be used as a diagnostic method for sensitivity to any allergens from different sources in vitro.

## Introduction

Allergic respiratory diseases are a growing health problem worldwide, affecting 30% of the population [1]. Asthma, allergic rhinitis, and chronic rhinosinusitis are induced by inhalation of aeroallergens such as pollen. The pathophysiology of allergic diseases is influenced by the binding of allergen-IgE by the high-affinity IgE receptor (FcεRI) on the surface of mastocytes and basophils. These cells release preformed inflammatory mediators involved in the allergy symptoms, such as histamine, prostaglandin, thromboxanes, and leukotrienes [2].

In clinical practice, the skin prick test (SPT) is the gold standard test for allergy diagnosis. However, the interpretation of results may vary depending on the allergenic extract used, sometimes leading to misdiagnoses [3]. An alternative to evaluate the allergens in vitro is the basophil activation test (BAT). The BAT is a functional assay that measures the degree of basophil degranulation following stimulation with allergens by flow cytometry [4], and it has been used successfully in respiratory allergic disease and food and Hymenoptera diagnosis. Another alternative to evaluate allergic reactions in vitro is to use immortalized cells, such as rat basophilic leukemia (RBL) cells.

RBL cells, obtained in the 70s, have been employed to understand the degranulation process [5], mainly the RBL-2H3 line. These cells were transfected with the three subunits (α, β, and γ) of the human FcεRI receptor; however, the α-chain is the minimal subunit required for human IgE recognition. Therefore, cell lines such as SX-38, RBL30/25, RBL48, NFAT-DsRed, and RS-ATL8 have been generated for allergy testing [6]. Nevertheless, a major limitation is that these cell lines are not commercially available in many countries because of customs regulations.

In this study, we developed an RBL-2H3 cell model expressing the human FcεRIα receptor and a degranulation reporter gene as a complementary in vitro test for allergic reactions with pollen proteins and sera from sensitive patients.

## Methods

### Plasmid selection

Plasmids that encode the α-chain of the human FcεRI receptor were searched in the commercial plasmid database. We chose the hFcεRIα plasmid from Origene (RC203321), which has a Kan/Neo resistance marker for selecting transfected cells. Additionally, we selected the pSIRV-NF-kB-eGFP plasmid from Addgene (118093), which has the promotor of the transcription factor NF-kB and the reporter gene GFP.

### Cell culture and transfection

RBL-2H3 wild-type cells (ATCC, CRL-2256) were cultivated in DMEM medium (Gibco, 31600034) supplemented with 10% serum fetal bovine (Gibco, A47668-01) at 37 °C with 5% CO_2_. For transfection, 1500 ng of plasmid pSIRV-NF-kB-eGFP and 500 ng of hFcεRIα were diluted in 250 µL Opti-MEM 1X (Gibco, 31985062). Then, RBL-2H3 cells were incubated for 24 h with the lipotransfection mix (OriGene, TurboFectin 8.0, TF81001) and 600 µg/mL of G418 (InvivoGen, ant-gn-5) for selection.

### Extracting pollen proteins from *Ligustrum lucidum*

A 50 mg of *L. lucidum* pollen was suspended in 1 mL of cold precipitate solution (20% TCA and 0.2% DTT) and then disrupted with a homogenizer (Wheaton). The extract was incubated for 1 h at − 20 °C and centrifuged for 13,000 × *g* for 20 min at 4 °C. Proteins were washed twice with 1 mL of cold acetone with 0.2% DTT. Then, the pellet was dried at room temperature and resuspended in 600 µL of rehydration buffer (7 M urea, 2 M thiourea, 4% CHAPS, and 40 mM DTT). Total protein concentration was determined by Bradford assay (Sigma, SLCG1879), and protein integrity was evaluated on 13% polyacrylamide SDS-PAGE gels stained with Coomassie blue.

### Selection of sera from allergic patients

Sera of four patients diagnosed with respiratory allergies sensitized to *Ligustrum* and four healthy volunteers were recruited in the outpatient allergy clinic of the Instituto Nacional de Enfermedades Respiratorias (INER) in Mexico City. The human ethics and research committee of INER approved the protocol for serum sampling with the number B07-21. The research was conducted according to the 1975 Declaration of Helsinki (as revised in 1983), which is consistent with Good Clinical Practice Guidelines.

### Reporter assay

RBL-2H3 transfected were cultivated in DMEM medium (Gibco, 31600034) supplemented with 10% serum fetal bovine (Gibco, A47668-01) and with 150 µg/mL of G418 (InvivoGen, ant-gn-5) at 37 °C with 5% CO_2_. Cells were trypsinized, seeded in 96-well cell culture plates, and grown in the same conditions until a confluence near 60% was reached. Cells were incubated for 24 h with the patient´s sera at 1:100 in a final volume of 100 µL of the culture medium. Later, cells were washed with PBS 1x and incubated with 0, 10, and 100 µg/mL of protein extracts for 3 h. A healthy patient’s sera (HP) 1:100 and 100 µg/mL of pollen proteins were used for negative control. Compound 48/80 (Sigma, C2313) was used as a positive control at 100 µg/mL. Cells were observed under fluorescent microscopy. Experiments were done in triplicates.

## Results

**Fig. 1:**
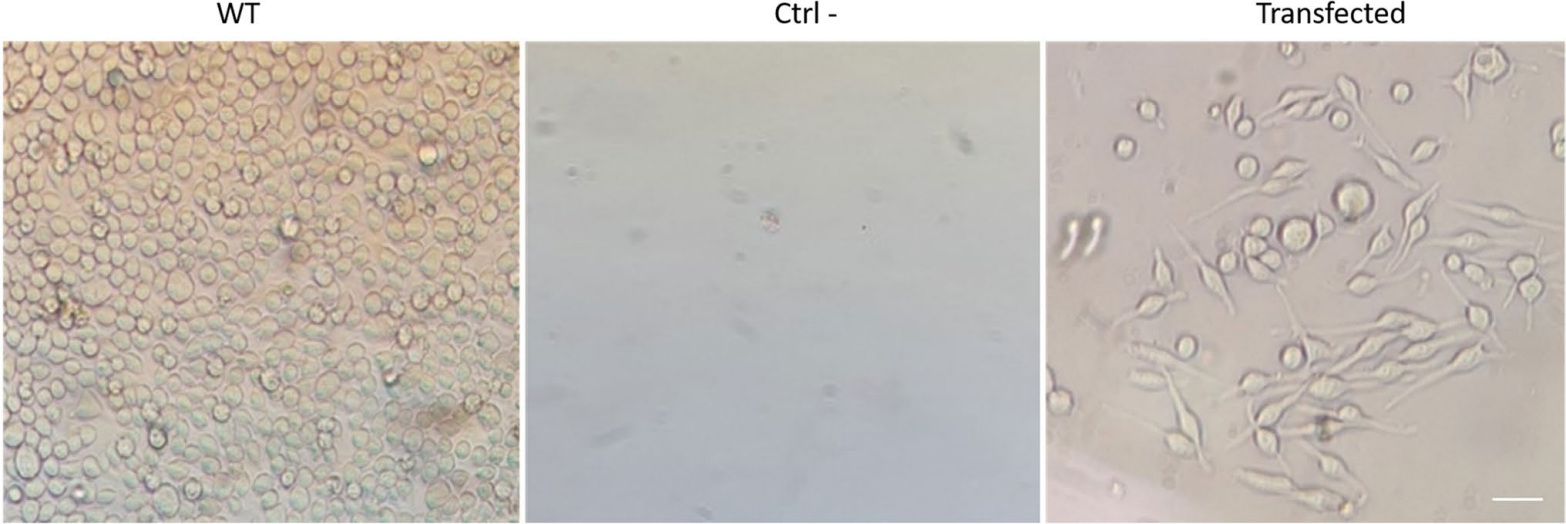
Selection of transfected RBL-2H3 cells. Wild-type RBL-2H3 cells were cultured without G418 as a control (WT); WT RBL-2H3 cells (Ctrl -) and transfected cells (transfected) were cultured with 600 µg/mL of G418 for 7 days. Scale bar = 20 µm.

To establish a cell line expressing the α-chain of the human FcεRI receptor and reporter gene, we transfected the hFcεRIα vector that also contains a Kan/Neo resistance marker for selecting transfected cells. The second vector selected was the pSIRV-NF-kB-eGFP, which contains the reporter gene GFP with the promotor of the transcription factor NF-kB. RBL-2H3 cells transfected with both plasmids were obtained after 7 days when WT cells were completely dead by G418 (Fig. 1). The cultures were reseeded every 2–3 days with G418 to maintain selective pressure. PCR confirmed the integration of plasmids into genomic DNA (data not shown).

**Fig. 2:**
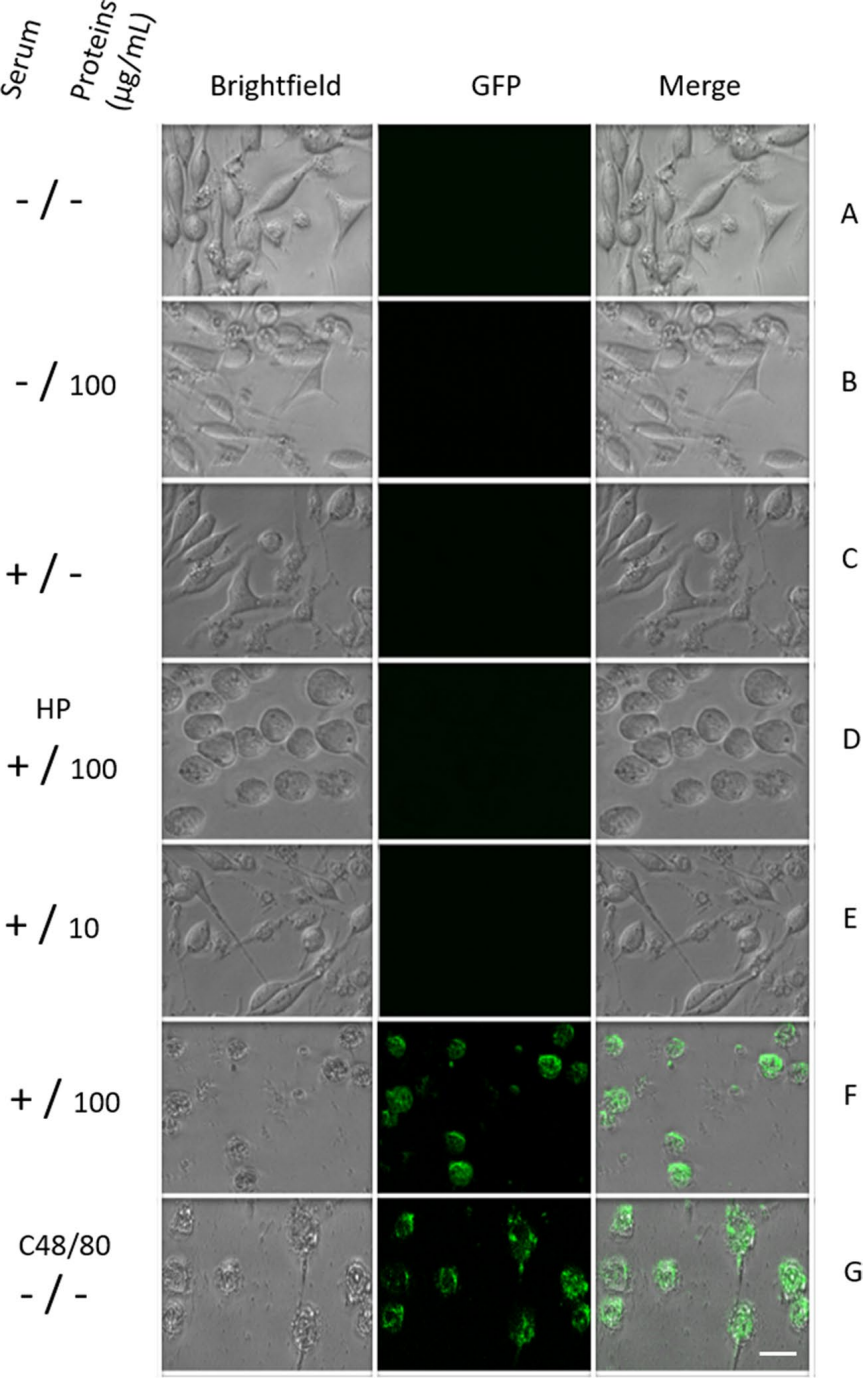
GFP signals in RBL-2H3 cells with different treatments. Transfected cells were treated without sera and proteins (**A**) and with sera and proteins alone (**B–C**). Stimulated with sera from healthy patients (HP) and proteins (**D**). Different protein concentrations were employed with sera from allergic patients (**E–F**). Positive control compound 48/80 (**G**). After stimulations, cells were observed under a fluorescent microscope. Scale bars = 10 µm

To evaluate the capacity of this model to detect allergens in vitro, we used proteins from *Ligustrum lucidum* pollen and sera from allergic patients. The high sera dilution, 1:100, was the best concentration in which the cells responded (data not shown). We incubated RBL-2H3 transfected cells with patients’ sera at 1:100 and with pollen protein extracts. All the time, G418 was maintained to the culture medium. Transfected cells without sera and proteins were included as controls to determine whether these cannot activate the cells (Fig. 2A–C). In addition, one culture was treated with sera from healthy patients (HP) and proteins (Fig. 2D), and one with compound 48/80 as a positive control (Fig. 2G). Two cultures were stimulated with patients’ sera at different concentrations of pollen proteins (Fig. 2E–F).

We did not observe any signal of GFP in RBL-2H3 transfected without sera and proteins (Fig. 2A) and with sera or proteins alone (Fig. 2B–C). Neither any critical change in their morphology was detected. When cells were treated with sera from HP and proteins, GFP was not observed, but morphology changed (Fig. 2D). Unlike when RBL-2H3 cells were stimulated with sera 1:100 and 10 µg/mL of pollen protein, few cells showed a GFP signal and some changes in their morphology (Fig. 2E). Interestingly, GFP signal and morphology changes are more notorious when cells are treated with sera 1:100 and 100 µg/mL of pollen proteins (Fig. 2F) and when compound 48/80 is added (Fig. 2G). In these cells, we can appreciate changes in the cellular shape, appearing rounded with cytoplasmic granules.

**Fig. 3:**
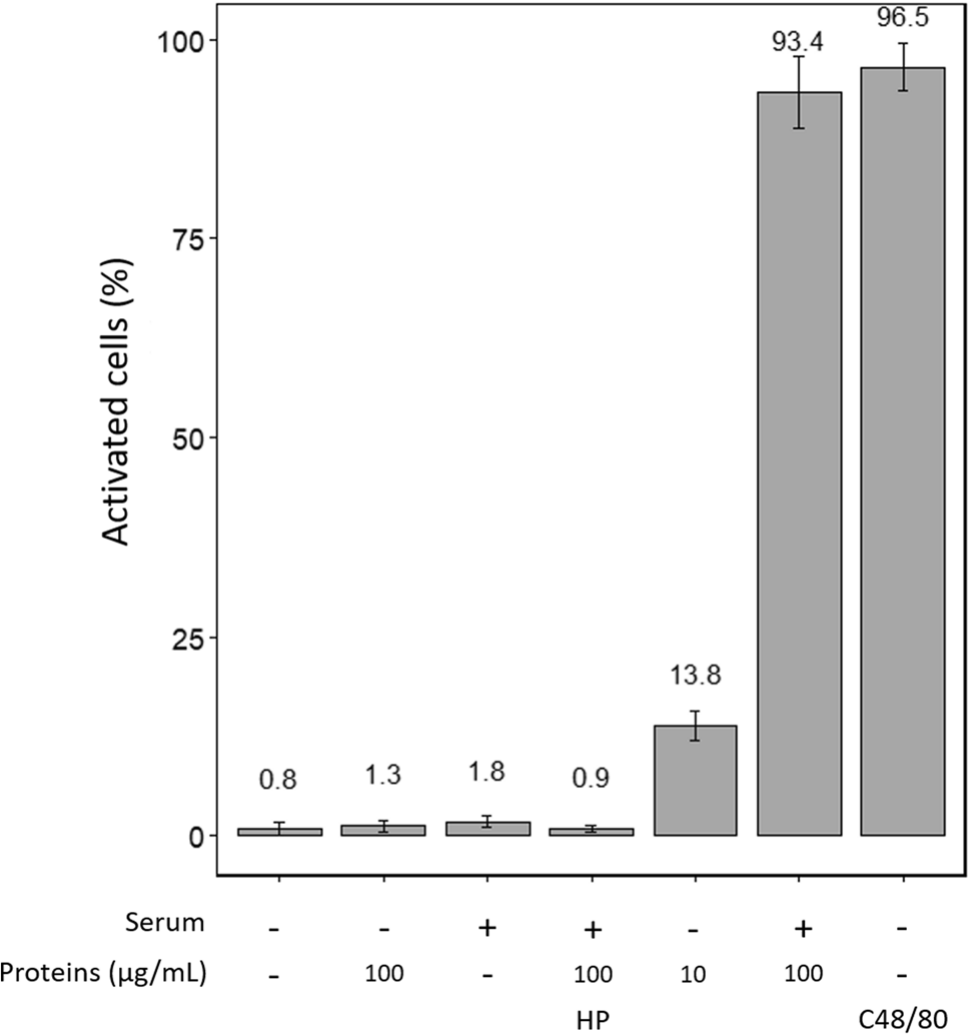
Effects of stimulation on activation in RBL-2H3 cells. Cells were treated with proteins from the pollen of *L. lucidum* (10 and 100 µg/mL), followed by antigen stimulation (1:100). Compound 48/80 was used as a positive control, and sera from healthy patients as a negative control (HP). Activation was determined by counting the cell number with a GFP signal. Data are normalized to C48/80

We repeated these experiments and counted the number of GFP-positive cells for three biological experiments. Results were normalized with the positive control C48/80 (Fig. 3). With RBL-2H3 transfected alone, with only serum or proteins, and the control with healthy patients, the percentage of positive cells was around 0.8 to 1.8%. This background may be a result of the apoptotic cells. We also notice that high-density cultures activate without stimulation (data not shown).

The percentage of cellular activation was close to C48/80 with RBL-2H3 transfected and stimulated with sera 1:100 and 100 µg/mL of pollen proteins. Meanwhile, the RBL cells stimulated with the same concentration of serum and 10 µg/mL of proteins only reached 13.8% activation.

## Discussion

Respiratory allergic diseases are increasing worldwide with air pollutants and climate change playing an important role in the severity of asthma and allergic rhinitis. Therefore, continuous characterization of allergens and the development of diagnostic allergy tests are necessary for better diagnosis. Although the SPT is a good diagnostic test, interpretation is often difficult because sensitization and clinical history do not coincide, and there is a risk of anaphylaxis in some cases. An alternative is in vitro IgE tests that use cells such as BAT or RBL. Previously, RBL cell lines have been developed and successfully used in allergic tests. However, the problem is that these cell lines are not commercially available. Hence, the present study aims to develop a cellular model with immortal RBL-2H3 cells as an alternative and complementary method to diagnose allergies in vitro.

Since RBL cells are derived from leukemia rats, human IgE does not recognize their IgE receptor. As a result, several groups have cloned the three subunits of human IgE receptors in RBL cells with different expression levels [6]. Nevertheless, Taudou and colleagues reported that the α-chain is the minimal subunit needed in RBL cells to be sensitized to human IgE [7]. In this sense, we used a commercial plasmid that encodes the α-chain of the human FcεRI receptor with the resistance gene to G418 to humanize RBL-2H3 cells. It is crucial to assess the activation potential of these cells, and employing reporter genes offers significant benefits compared to biochemical assays. Kalli and colleagues have used an RBL cell line that uses the nuclear factor of activated T cells (NFAT) a responsive luciferase reporter gene [8]. Luciferase can be measured using appropriate chemiluminescent substrates. This process is rapid and more sensitive than measuring beta-hexosaminidase release when RBL cells are activated. Then, the plasmid pSIRV-NF-kB-eGFP was selected because it contains a GFP reporter gene under activation of the NF-kB transcription factor. The activation of NF-kB signaling in immune cells drives the expression of pro-inflammatory cytokinesassociated with allergic diseases [9].

Transfected cells were tested with the sera of allergenic patients to *Ligustrum* tree pollen. Interestingly, in 2015, our group identified six IgE-binding proteins in this pollen employing an immunoproteomics approach. Indeed, 2-DE immunoblotting and mass spectrometry allowed the  identification of the following allergens: profilin, enolase, β-1,3-glucanase, polygalacturonase, alanine aminotransferase, and two beta subunits of ATP-synthase [10]. A limitation is that using a pool of patients’ sera and pollen proteins makes it impossible to determine what each patient is allergic to. Nevertheless, at this time, employing these sera and the proteins, we found that the method we developed is a suitable assay for detecting allergen-specific IgE antibodies. As reported in similar experiments, we employed two sera dilutions, 1:50 and 1:100, with high protein concentrations [11]. Cells responded better with the diluted sera concentration. This could be explained because of the cytotoxicity of the sera over RBL cells, which can be due to serum components such as activated complement. Higher dilutions are recommended but can be insufficient to sensitize cells. Our results showed that cells are neither activated by themselves nor employing sera derived from healthy patients. Likewise, sera and proteins alone cannot stimulate RBL-2H3 cells. Spontaneous degranulation near 1% was observed with the negative controls; we hypothesized that this observation could be due to incubation with sera alone or by senescent cells. Cellular senescence-associated changes can lead to mast cell degranulation (IgE-independent) [12].

This model was activated at levels near positive control using sera 1:100 and 100 µg/mL of pollen proteins. Compound 48/80 has been reported as an activator of degranulation in mast cells [13]. When RBL were stimulated with the same sera concentration and 10 µg/mL of pollen proteins, cell activation was seven-fold lower than that caused by C48/80. These results show that both sera and protein concentrations mediate cellular activation, and GFP is expressed as a result of cell activation. Of particular interest was the finding that changes in cellular shape and granule appearance were observed upon RBL cell activation. These granules  indicate basophil degranulation by IgE binding to the FcεRI receptor. The cellular changes could be taken as a parameter of cell activation without using a fluorescent microscope. The advantages of working with RBL cells lie in their easy cultivation and the ability to test them with various sera and allergenic sources. Indeed, the cellular model described in the present manuscript can be established using commercially available plasmids, allowing its implementation in laboratories with limited infrastructure, as it does not require the use of a flow cytometer. Additionally, integrating  a GFP-ELISA kit could enhance the  accuracy of the results.

The major challenge in allergy diagnosis is the creation of accessible and dependable diagnostic methods that enable accurate prediction. In this study, we have shown that integrating reporter genes into humanized RBL cells could emerge as a potent tool for the clinical diagnosis of intricate allergic conditions and evaluating innovative anti-allergic medications.

## Data Availability

Not applicable.
